# Sleep, 24-Hour Activity Rhythms, and Subsequent Amyloid-β Pathology

**DOI:** 10.1001/jamaneurol.2024.1755

**Published:** 2024-06-24

**Authors:** Phuong Thuy Nguyen Ho, Sanne J. W. Hoepel, Maria Rodriguez-Ayllon, Annemarie I. Luik, Meike W. Vernooij, Julia Neitzel

**Affiliations:** 1 Department of Radiology and Nuclear Medicine, Erasmus University Medical Centre, Rotterdam, the Netherlands; 2 Department of Epidemiology, Erasmus University Medical Centre, Rotterdam, the Netherlands; 3 Trimbos Institute—the Netherlands Institute of Mental Health and Addiction, Utrecht, the Netherlands; 4 Department of Epidemiology, Harvard T. H. Chan School of Public Health, Boston, Massachusetts

## Abstract

**Importance:**

Sleep disturbances are common among older adults and have been associated with the development of Alzheimer disease (AD), such as amyloid-β (Aβ) pathology. For effective AD prevention, it is essential to pinpoint the specific disturbances in sleep and the underlying 24-hour activity rhythms that confer the highest risk of Aβ deposition.

**Objective:**

To determine the associations of 24-hour activity rhythms and sleep with Aβ deposition in adults without dementia, to evaluate whether disrupted 24-hour activity and sleep may precede Aβ deposition, and to assess the role of the apolipoprotein E ε4 (*APOE4*) genotype.

**Design, Setting, and Participants:**

This was an observational cohort study using data from the Rotterdam Study. Of 639 participants without dementia who underwent Aβ positron emission tomography (PET) from September 2018 to November 2021, 319 were included in the current study. Exclusion criteria were no *APOE* genotyping and no valid actigraphy data at the baseline visits from 2004 to 2006 or from 2012 to 2014. The mean (SD) follow-up was 7.8 (2.4) years. Data were analyzed from March 2023 to April 2024.

**Exposures:**

Actigraphy (7 days and nights, objective sleep, and 24-hour activity rhythms), sleep diaries (self-reported sleep), Aβ42/40, phosphorylated tau (p-tau)_181_ and p-tau_217_ plasma assays, ^18^F-florbetaben PET (mean standard uptake value ratio [SUVR] in a large cortical region of interest), and *APOE4* genotype.

**Main Outcomes and Measures:**

Association of objective and self-reported sleep and 24-hour activity rhythms at baseline with brain Aβ PET burden at follow-up.

**Results:**

The mean (range) age in the study population was 61.5 (48-80) years at baseline and 69.2 (60-88) years at follow-up; 150 (47%) were women. Higher intradaily variability at baseline, an indicator of fragmented 24-hour activity rhythms, was associated with higher Aβ PET burden at follow-up (β, 0.15; bootstrapped 95% CI, 0.04 to 0.26; bootstrapped *P* = .02, false discovery rate [FDR] *P* = .048). *APOE* genotype modified this association, which was stronger in *APOE4* carriers (β, 0.38; bootstrapped 95% CI, 0.05 to 0.64; bootstrapped *P* = .03) compared to noncarriers (β, 0.07; bootstrapped 95% CI, −0.04 to 0.18; bootstrapped *P* = .19). The findings remained largely similar after excluding participants with AD pathology at baseline, suggesting that a fragmented 24-hour activity rhythm may have preceded Aβ deposition. No other objective or self-reported measure of sleep was associated with Aβ.

**Conclusions and Relevance:**

Among community-dwelling adults included in this study, higher fragmentation of the 24-hour activity rhythms was associated with greater subsequent Aβ burden, especially in *APOE4* carriers. These results suggest that rest-activity fragmentation could represent a modifiable risk factor for AD.

## Key Points

**Question:**

Are disturbances of 24-hour activity rhythms and sleep associated with subsequent brain amyloid-β deposition?

**Findings:**

In this cohort study of 319 adults aged 48 to 80 years without dementia, a more fragmented 24-hour activity rhythm was associated with higher amyloid-β burden assessed 7.8 years later, and this association was stronger in Alzheimer disease (AD) genetic risk (apolipoprotein E4) carriers compared to noncarriers. Accounting for AD pathology at baseline did not notably change our findings.

**Meaning:**

The findings suggest that rhythm disturbances can precede amyloid-β deposition and may be a modifiable risk factor for AD.

## Teaser

This cohort study evaluates associations of sleep and 24-hour activity rhythms with amyloid-β deposition.

## Introduction

Considering the increasing prevalence of Alzheimer disease (AD), it is crucial to identify modifiable risk factors.^1,2^ Disturbances of sleep and the underlying day-night (24-hour) activity rhythms may be actionable risk factors for AD.^3,4,5^ There is emerging evidence connecting disrupted sleep and 24-hour activity rhythms to amyloid-β (Aβ) accumulation, one of the defining pathologies of AD.^6,7^ A major discovery was that the clearance of Aβ from the brain through the glymphatic system is sleep-dependent and occurs twice as fast in sleep than in wake.^8,9^ To develop effective prevention, it is essential to determine which specific aspects of sleep and 24-hour activity rhythms are associated with Aβ deposition in the absence of dementia.

The sleep measures studied so far in relation to Aβ pathology fall into 4 broad categories: increased or decreased sleep duration,^10,11,12,13,14,15,16,17,18^ poor sleep quality,^13,15,17,18,19,20^ daytime sleepiness,^10,12,14,21^ and disturbances of the 24-hour activity rhythms.^7,16,22^ However, a problem with existing research is that different self-reported and objective measures have been used, leading to conflicting results. For example, shorter self-reported sleep duration was associated with increased Aβ burden in most studies.^11,12,13,14^ In contrast, this association was not confirmed in studies using an objective estimate of sleep duration based on actigraphy,^15,17,18^ a validated tool that estimates sleep and wake based on movements of the wrists.^23^ To our knowledge, no study has examined how both objective and self-reported sleep estimates relate to Aβ pathology, while the only 2 actigraphy studies exploring the connection between altered 24-hour activity rhythms and Aβ levels reported inconsistent associations.^22,24^

Two factors further complicate the interpretation of previous research. First, the association between sleep, 24-hour activity rhythms, and Aβ pathology is probably bidirectional.^25,26,27,28^ Since most previous studies measured sleep, 24-hour activity rhythms, and Aβ around the same time^11,12,15^ or did not disclose this information,^14,17,18,19^ it is difficult to conclude whether the identified disturbances are a risk factor that would be worth considering in a prevention trial or the result of already developed Aβ pathology. Second, most previous studies did not investigate an effect modification by apolipoprotein E (*APOE*) genotype.^11,12,17,18,19,21,29^ In AD mouse models, chronic sleep deprivation increased Aβ plaques, but only when they expressed human *APOE4*, not *APOE3*.^30^ Previous studies in humans may have underestimated the aversive effect of disturbed sleep and 24-hour activity rhythms in *APOE4* risk carriers.^11,12,17,18,19,21^

In the current study, we assessed sleep and 24-hour activity rhythms in 319 participants without dementia from the prospective population-based Rotterdam Study. About 8 years later, we assessed Aβ burden by positron emission tomography (PET). We addressed limitations of former studies by investigating a broad range of both objective and self-reported sleep and 24-hour activity rhythms measures and investigated a potential interaction with *APOE4*. To infer whether identified disturbances were likely a risk factor or result of Aβ pathology, we accounted for AD pathology at baseline.

## Methods

This study was reported according to the Strengthening the Reporting of Observational Studies in Epidemiology (STROBE) reporting guideline. The Rotterdam Study was approved by the Medical Ethics Committee of the Erasmus University Medical Center and by the Dutch Ministry of Health, Welfare and Sport. All participants provided written informed consent before participation.

### Setting

**Figure 1.**
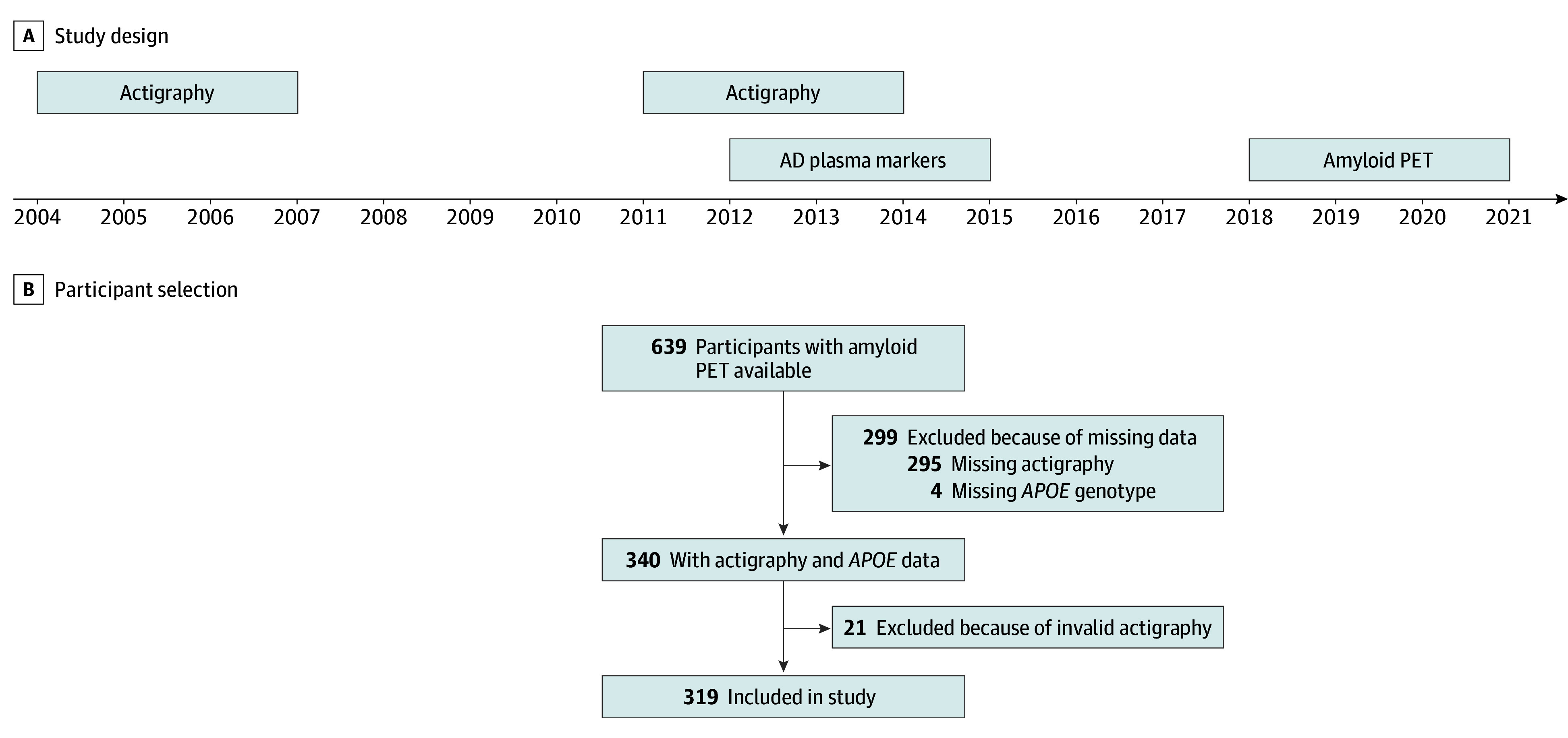
Study Design and Participant Selection A, Diagram of the relevant measurements in the Rotterdam Study (RS). The current study included participants from the second and third RS cohort (RS-II, RS-III). Participants are reexamined on average every 4 years.^31,32^ Actigraphy and sleep diary were collected in the visits from 2004 to 2007 (RS-II-2 and RS-III-1) and from 2011 to 2014 (RS-II-3 and RS-III-2). Alzheimer disease plasma markers were measured in blood samples collected between 2012 to 2015. A subgroup of RS-II and RS-III participants were invited to undergo amyloid positron emission tomography (PET) during an extra visit from 2018 to 2021.^10,33^ B, Of 2068 eligible individuals for PET, 1697 were invited, 645 (response rate 38%) made an appointment, and 639 amyloid PET scans were acquired. A total of 340 participants had *APOE* genotyping and actigraphy data. Twenty-one had to be excluded due to invalid actigraphy measurements, including malfunctioning, measurements taken during the change to or from daylight savings time, and less than 96 hours of consecutive recording. The final sample included 319 participants. If participants had 2 valid actigraphy measurements, we included the most recent one (n = 42 from 2004-2007; n = 277 from 2011-2014).

The current study was embedded in the Rotterdam Study, a prospective population-based study located in the district of Ommoord, Rotterdam, the Netherlands. Originally started in 1990 with 7983 participants (RS-I), the study expanded with more participants in 2000 (3011 participants [RS-II]) and 2006 (3932 participants [RS-III]). Participants are reexamined on average every 4 years.^31,32^ From December 2004 to April 2007 and from February 2011 to June 2014, a subsample of participants was invited to participate in the actigraphy study. This is the baseline of the current investigation. Between September 2018 and November 2021, a subsample of RS-II and RS-III participants were invited to undergo a PET examination (Figure 1A). Eligibility criteria were ≤60 years old, brain magnetic resonance imaging between 2011 and 2016, no PET-related contraindications, and no clinical diagnosis of dementia.^10,33^ Of the 639 participants in the PET study, 340 had actigraphy. We excluded participants without *APOE* (n = 4) or valid actigraphy data (n = 21). Our final sample consisted of 319 participants (Figure 1B).

### Sleep and 24-Hour Activity Rhythms

#### Objective Measures

Participants wore an actigraph, either an Actiwatch 4 (Cambridge Technology) or a GeneActiv (Activinsights) on the nondominant wrist for 7 consecutive days and night. A validated algorithm ensured compatibility between the data from the 2 actigraphs.^34^ Participants pressed a marker button on the device to denote bedtime and getting up time. Within this defined time, sleep and wakefulness were estimated using a validated algorithm against polysomnography with a threshold of 20 activity counts.^35^ Counts were summed per 30-second epochs.

We obtained 4 measures of objectively estimated sleep. Total sleep time (hours) was defined as the time between sleep start (first immobile period of at least 10 minutes after bedtime) and sleep end (last immobile period of at least 10 minutes before getting up time), minus the time classified as awake by the algorithm. Sleep onset latency (minutes) was calculated as the time between bedtime and sleep start. Sleep efficiency (%) was calculated as total sleep time / time in bed × 100. Wake after sleep onset (minutes) was calculated as the time scored as wake between sleep start and end.

**Figure 2.**
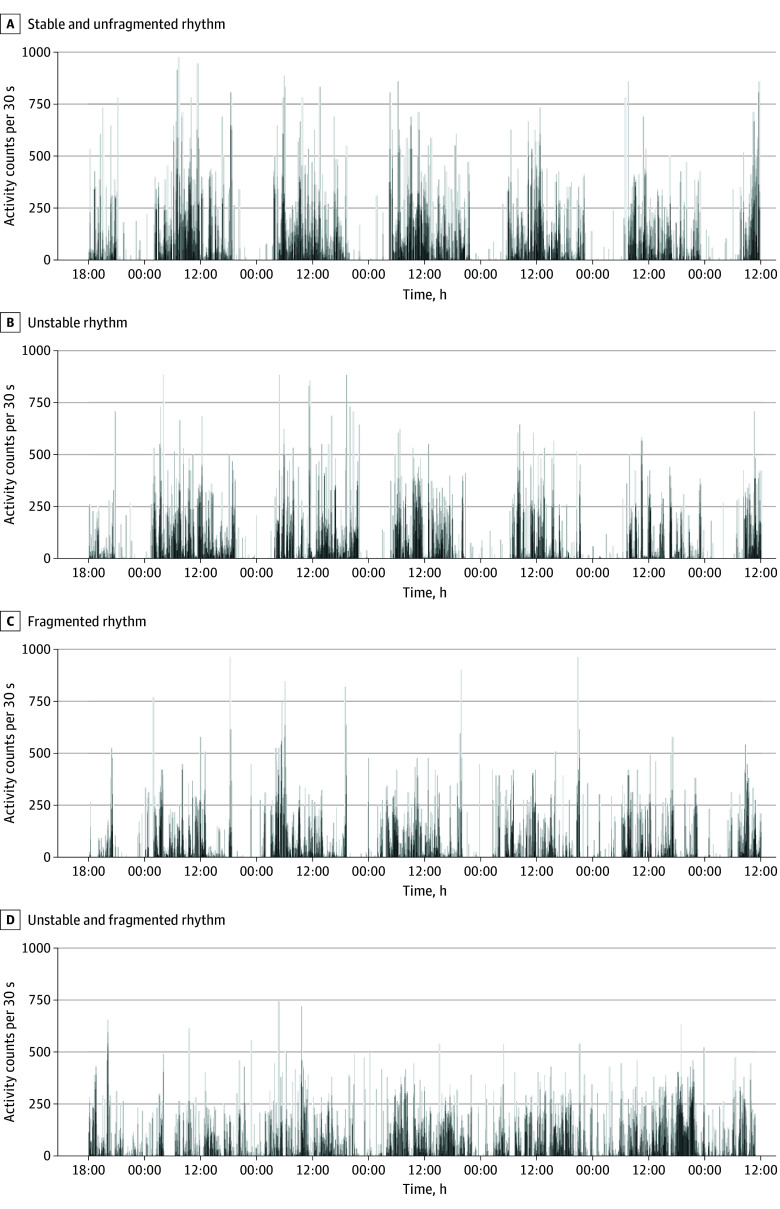
Examples of High and Low Interdaily Stability and Intradaily Variability Four examples of 24-hour rest and activity rhythms are shown. The x-axis represents time (0:00, midnight; 12:00, noon), and the y-axis represents activity counts per 30 seconds. Participants started wearing the actigraph unit at 18:00 hours on day 1. These activity counts are scaled relative to the individual means and cannot be compared easily across persons. A, Rhythm with high interdaily stability and low intradaily variability. B, Rhythm with low interdaily stability and low intradaily variability. C, Rhythm with high interdaily stability and high intradaily variability. D, Rhythm with low interdaily stability and high intradaily variability.

We further obtained 3 measures of 24-hour activity rhythms. Interdaily (day-to-day) stability was calculated as the ratio of the variance of the average activity patterns around the mean and the overall variance.^36^ Lower values reflect a more unstable rhythm (Figure 2). Intradaily variability measured the within-day fragmentation of the rhythms which was calculated as the ratio of the mean squares of the differences between all successive hours and the mean squares around the grand mean.^36,37^ Higher values reflect a more fragmented rhythm (Figure 2). Both lower interdaily stability and higher intradaily variability have been associated with poor health and higher mortality risk.^37,38^ L5 start time is the hour of the day marking the start of the five consecutive hours with lowest activity.

#### Self-Reported Measures

Each participant kept a daily sleep diary along with actigraphy. We obtained 7 measures of self-reported sleep.^39^ Daily total sleep time (hours), sleep onset latency (minutes), and bedtime and get-up time were averaged over the week. Time in bed (hours) was calculated as the difference between bedtime and getting-up time; missing times were imputed from the actigraphy marker participants pressed when going to bed and getting up. Sleep efficiency (%) was calculated from duration and time in bed. Sleep quality was calculated as the summed daily average of three questions, eg, “Do you think you slept well last night?” Napping was measured as the average number of days per week participants took one or more naps before 18:00. Daytime sleepiness was measured as number of days per week that participants felt sleepy or tired during the day.

### Amyloid PET Acquisition and Processing

^18^F-florbetaben amyloid PET imaging was performed and processed according to an established pipeline.^10,33^ In short, PET images were obtained on a Siemens Biograph mCT PET/CT (Siemens Healthineers) 90 to 110 minutes after intravenous injection of 300 MBq (±20%) of the ^18^F-florbetaben tracer (Neuraceq; Life Molecular Imaging GmbH). We quantified participants’ Aβ burden using the average cortical standard uptake value ratio (SUVR), that is, tracer uptake in frontal, cingulate, lateral parietal, and lateral temporal regions divided by uptake in the cerebellar reference region.^40^

### AD Pathology at Baseline

As we did not collect Aβ PET at baseline (when actigraphy was collected), we decided to infer the burden of AD pathology based on AD plasma markers collected at baseline. In short, plasma samples (n = 313) were analyzed at the Neurochemistry Laboratory, Amsterdam. The Simoa Neuro 4-Plex E Kit was used to measure Aβ40 and Aβ42. The Simoa pTau-181 Advantage V2.1 Kit and ALZpath pTau-217 CARe Advantage Kit were used to measure phosphorylated tau (p-tau) at codon 181 (p-tau_181_) and 217 (p-tau_217_). The time between actigraphy and plasma sampling was a mean (SD) 11 (2) months. We assumed that normal plasma values collected after actigraphy were also normal at the time of actigraphy.

### Statistical Analyses

All analyses were performed using R version 4.1.2 (R Foundation). Sleep and 24-hour activity rhythm variables were standardized and winsorized to 4 standard deviations. SUVR was log-transformed and standardized. We used diagnostic plots to ensure that linear regression assumptions were met. As SUVR values are inherently right-skewed and therefore error terms were not normally distributed, we calculated bootstrapped confidence intervals and *P* values (eMethods 1 in Supplement 1). To correct for multiple comparisons, we reported false discovery rate (FDR) *P* values.

Linear regression models were used to determine the association between sleep, 24-hour activity rhythms at baseline and Aβ PET SUVR at follow-up. To control for potential confounders (eMethods 2 in Supplement 1), we included age (years), sex (female/male), carrying at least 1 *APOE*4 allele (yes/no), time between PET and actigraphy (years), and type of actigraph (Actiwatch or GeneActiv) (model 1). We additionally controlled for education (primary, lower, intermediate, and higher), self-reported possible sleep apnea (yes/no), self-reported sleep medication (yes/no), body mass index, hypertension (yes/no), diabetes (yes/no), current smoking (yes/no), physical activity (metabolic equivalents hours/week), depressive symptoms (0-60), and paid employment status (yes/no) (model 2). In sensitivity analyses, we applied a rank-based inverse normal transformation (RankNorm function, RNOmni R package version .1.0.1.2) of the SUVR values, which resulted in normally distributed regression error terms. We further estimated how much the actigraph device influenced our results by excluding participants who were measured with the GeneActiv watch. To test potential nonlinear associations with Aβ pathology, we added a quadratic term of total sleep time, time in bed, and L5 start time. As only 3 participants had a total sleep time of more than 8 hours,^41^ we could not assess U-shaped associations.^12^ We also performed logistic regression with amyloid PET status as the outcome.

We further tested the potential effect modification of *APOE4* on the association between sleep, 24-hour activity rhythms, and Aβ pathology. Specifically, we modeled product terms of each sleep and 24-hour rhythm measure with *APOE4* (ie, multiplicative interactions) in separate linear regression models. In case of a significant interaction, we ran stratified analyses for *APOE4* carriers and noncarriers.

We next investigated whether disturbances of sleep and 24-hour activity rhythms were a risk factor or result of Aβ pathology using 2 complementary approaches. First, we statistically controlled for the burden of AD pathology at baseline by including AD plasma marker levels (Aβ42/40, p-tau_181_, and p-tau_217_) as covariates. Second, we excluded participants with AD pathology at baseline, hypothesizing that if sleep and 24-hour activity rhythm disturbances preceded Aβ pathology, then the association should remain. We excluded 5%, 10%, and 15% of the most abnormal plasma values; 15% was chosen as the highest exclusion threshold because the prevalence of a positive Aβ PET scan at follow-up in our sample was 15.4%. We additionally applied an established p-tau_217_ cut point of 0.63 pg/mL, previously optimized by Ashton et al^42^ to detect amyloid PET positivity in 3 independent cohorts. Two-tailed *P* values less than .05 were considered significant.

## Results

### Sample Characteristics

The sample included 319 participants (150 [47% female) with a mean (SD) age of 61.5 (5.4) years at the baseline sleep assessment and 69.2 (5.3) years at the PET acquisition (Table 1). The mean (SD) follow-up time was 7.8 years (2.4). A total of 90 participants (28.2%) were *APOE4* carriers, and 49 participants (15.4%) had a positive Aβ status. eTable 1 in Supplement 1 presents the demographic characteristics stratified by *APOE4* carriership. Correlations between sleep and 24-hour activity rhythm measures are shown in eFigure 1 in Supplement 1.

**Table 1.** Sample Characteristics

Variable	Overall, No. (%)
No.	319
Demographic characteristics	
Age at sleep assessment, mean (SD), y	61.47 (5.40)
Age at PET, mean (SD), y	69.24 (5.25)
Years between sleep assessment and PET, mean (SD)	7.77 (2.38)
Sex	
Female	150 (47.0)
Male	169 (53.0)
Education	
Primary	21 (6.6)
Lower	89 (27.9)
Intermediate	87 (27.3)
Higher	122 (38.2)
Paid employment	
No	136 (49.8)
Yes	137 (50.2)
MMSE score at sleep assessment, mean (SD)	28.55 (1.31)
Genetic measures	
Ancestry	
African	2 (0.6)
Asian	4 (1.3)
European	280 (87.8)
Multiple	33 (10.3)
No. of *APOE4* alleles	
0	229 (71.8)
1	77 (24.1)
2	13 (4.1)
Amyloid PET measures	
Amyloid PET status	
Negative	270 (84.6)
Positive	49 (15.4)
SUVR, mean (SD)	1.03 (0.15)
24-h Activity rhythm measures from actigraphy, mean (SD)	
Interdaily stability	0.73 (0.11)
Intradaily variability	0.46 (0.14)
L5 start time, h:min	1:31 (1.19)
Objective sleep measures (actigraphy), mean (SD)	
Total sleep time, h	6.15 (0.86)
Sleep latency, min	17.49 (16.34)
Sleep efficiency, %	76.36 (7.91)
Wake after sleep onset, min	54.02 (22.46)
Subjective sleep measures (sleep diaries), mean (SD)	
Total sleep time—diary, h	6.73 (0.90)
Time in bed, h	8.07 (0.84)
Sleep latency, min	22.16 (19.95)
Sleep efficiency, %	83.80 (9.91)
Sleep quality	5.47 (1.56)
Napping	0.93 (1.56)
Daytime sleepiness, d/wk	0.66 (1.21)
Other	
Type of actigraph	
Actiwatch	253 (79.3)
GENEActiv	66 (20.7)
Sleep apnea	
No	243 (86.8)
Yes	37 (13.2)
Sleep medication	
No	282 (88.4)
Yes	37 (11.6)
BMI, mean (SD)	27.31 (3.87)
Hypertension	
No	141 (44.2)
Yes	178 (55.8)
Diabetes	
No	292 (91.8)
Yes	26 (8.2)
Smoking	
No	224 (80.6)
Yes	54 (19.4)
Physical activity, mean (SD), MET h/wk	59.67 (53.00)
Depressive symptoms, mean (SD), No.	4.27 (6.23)

Abbreviations: BMI, body mass index (calculated as weight in kilograms divided by height in meters squared); MET, metabolic equivalents; MMSE, Mini-Mental State Examination; PET, positron emission tomography; SUVR, standard uptake value ratio.

### Sleep and 24-Hour Activity Rhythms Associated With Aβ Pathology

**Figure 3.**
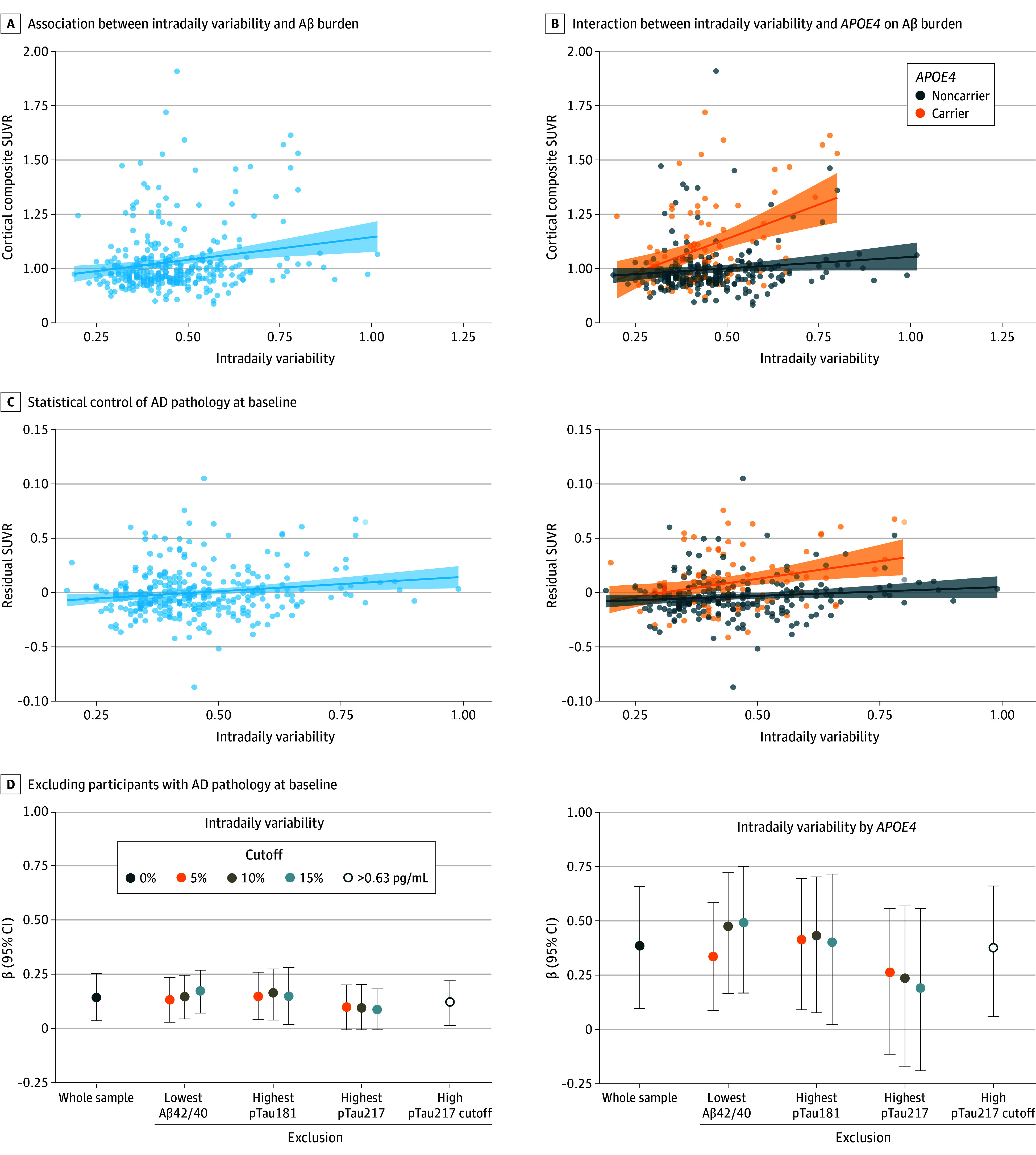
Association Between Fragmented 24-Hour Activity Rhythms at Baseline and Amyloid-β (Aβ) Positron Emission Tomography (PET) Burden at Follow-Up A, The scatterplot shows the association between intradaily variability (x-axis) and Aβ PET burden (y-axis) a mean (SD) 7.8 (2.4) years later measured as the mean cortical standard uptake value ratio (SUVR). The solid black line represents the linear regression line, with shaded areas indicating 95% CIs. B, The scatterplot displays the same association as in A separately for *APOE4* carriers (orange) vs noncarriers (gray). C, The scatterplots show the association between intradaily variability (x-axis) and residuals of Aβ PET SUVR (y-axis) after statistically controlling for (continuous values of) Aβ42/40, phosphorylated tau (p-tau)_181_, and p-tau_217_ at baseline. D, The graphs show the standardized β coefficients and bootstrapped 95% CIs (y-axis) of the main effect of intradaily variability (left graph) and the intradaily variability by *APOE4* interaction (right graph) on Aβ PET burden. To evaluate whether a fragmented 24-hour activity rhythm preceded Aβ deposition, we compared the effect size in the whole sample to those computed after excluding participants with the most abnormal Aβ42/40, p-tau_181_, or p-tau_217_ plasma levels at baseline (x-axis). We used 3 cut points for participant exclusion (color-coded). Note that 15% is a conservative threshold given that the prevalence of a positive Aβ PET scan at follow-up was 15.4%. Alternatively, we used an established p-tau_217_ cut point of >0.63 pg/mL.^42^

Higher intradaily variability, that is, stronger within-day fragmentation of the 24-hour activity rhythms, was significantly associated with more severe Aβ pathology (higher SUVR) after adjusting for age, sex, *APOE4* carriership, type of actigraphy device, and time between actigraphy and PET imaging (model 1: β, 0.15; bootstrapped 95% CI, 0.04 to 0.26; *P* = .007; bootstrapped *P* = .016, FDR *P* = .048) (Figure 3A). The effect size remained similar (although not significant after multiple test correction) when we additionally adjusted for sleep medication, education, possible sleep apnea, body mass index, hypertension, diabetes, smoking, physical activity, depressive symptoms, and employment status (model 2: β, 0.13; bootstrapped 95% CI, 0.03 to 0.25; *P* = .02; bootstrapped *P* = .03; FDR *P* = .12). Inverse normal transformation of SUVR values yielded similar results (model 1: β, 0.14; 95% CI, 0.04 to 0.25; *P* = .009). We also ensured that different actigraph devices did not influence our main finding (model 1 after excluding 66 datapoints from the GeneActiv watch: β, 0.20; bootstrapped 95% CI, 0.07 to 0.31; *P* = .001; bootstrapped *P* = .002). Logistic regression showed a trend level association between higher intradaily variability and a positive amyloid PET status (odds ratio [OR] 1.36; 95% CI, 0.94 to 1.93; *P* = .10) (eTable 2 in Supplement 1). No other objective measures were significantly associated with Aβ pathology, nor were any self-reported sleep measures (Table 2). We found no robust nonlinear association between total sleep time, time in bed, L5 start time, and Aβ pathology (eTable 3 in Supplement 1).

**Table 2.** Associations of 24-Hour Activity Rhythms, Sleep, and Amyloid-β (Aβ) Pathology

Variable	Missing data	Model 1				Model 2			
		β (95% CI)	*P* value	*P* value (boot)	*P* value (FDR)	β (95% CI)	*P* value	*P* value (boot)	*P* value (FDR)
24-h Activity rhythm measures (actigraphy)									
Interdaily stability	9	0.03 (−0.06 to 0.13)	.55	.49	.85	0.06 (−0.04 to 0.15)	.29	.23	.68
Intradaily variability	9	0.15 (0.04 to 0.26)	.007	.02	.05	0.13 (0.03 to 0.25)	.02	.03	.12
L5 start time	11	−0.01 (−0.11 to 0.09)	.85	.84	.85	−0.02 (−0.10 to 0.08)	.75	.74	.75
Objective sleep measures (actigraphy)									
Total sleep time	0	−0.10 (−0.19 to −0.01)	.06	.04	.20	−0.08 (−0.16 to 0.01)	.14	.09	.48
Sleep latency	0	−0.02 (−0.10 to 0.07)	.73	.70	.85	−0.03 (−0.13 to 0.06)	.62	.59	.72
Sleep efficiency	0	−0.04 (−0.14 to 0.05)	.45	.43	.85	−0.03 (−0.13 to 0.07)	.54	.53	.72
Wake after sleep onset	0	0.03 (−0.07 to 0.13)	.61	.63	.85	0.03 (−0.08 to 0.14)	.54	.57	.72
Subjective sleep measures (sleep diary)									
Total sleep time	0	−0.04 (−0.13 to 0.05)	.48	.40	.67	−0.03 (−0.11 to 0.06)	.61	.55	.72
Time in bed	0	−0.09 (−0.19 to 0.02)	.08	.09	.59	−0.07 (−0.17 to 0.03)	.18	.17	.72
Sleep latency	2	−0.02 (−0.12 to 0.09)	.76	.77	.76	0.00 (−0.10 to 0.12)	.95	.95	.95
Sleep efficiency	0	0.05 (−0.07 to 0.14)	.39	.36	.67	0.04 (−0.07 to 0.13)	.52	.46	.72
Sleep quality	0	0.04 (−0.05 to 0.12)	.45	.36	.67	0.04 (−0.06 to 0.12)	.52	.45	.72
Napping	0	−0.07 (−0.15 to 0.01)	.20	.14	.67	−0.06 (−0.16 to 0.02)	.23	.16	.72
Daytime sleepiness	0	0.02 (−0.06 to 0.10)	.69	.61	.76	0.03 (−0.06 to 0.12)	.62	.51	.72

### Modification Effect of APOE4

We found a significant interaction of fragmented 24-hour activity rhythms and *APOE4* on Aβ pathology (model 1: β, 0.38; bootstrapped 95% CI, 0.10 to 0.66; *P* = .002; bootstrapped *P* = .02; FDR *P* = .01; model 2: β, 0.37; bootstrapped 95% CI, 0.07 to 0.63; *P* = .003; bootstrapped *P* = .03; FDR *P* = .02). Stratified analysis showed that this association was stronger in *APOE4* carriers (β, 0.38; bootstrapped 95% CI, 0.05 to 0.64; *P* = .02; bootstrapped *P* = .03) than in noncarriers (β, 0.07; bootstrapped 95% CI, −0.04 to 0.18; *P* = .16; bootstrapped *P* = .19) (Figure 3B). The interaction effect was attenuated in the sensitivity analysis with inverse-normal-transformed SUVR values (model 1: β; 0.23; 95% CI, −0.01 to 0.48; *P* = .06). There were no other significant interactions (eTable 4 in Supplement 1).

### Fragmentation of the 24-Hour Activity Rhythms Likely Preceded Aβ Pathology

We used 2 approaches to test whether a fragmented 24-hour activity rhythm may have preceded Aβ pathology. First, we statistically controlled for AD pathology at baseline. Including plasma Aβ42/40, p-tau_171_, and p-tau_217_ levels in our regression model still yielded a robust association between fragmented 24-hour activity rhythms and Aβ pathology (β, 0.11; bootstrapped 95% CI, 0.02 to 0.20; *P* = .03; bootstrapped *P* = .03) (Figure 3C). The modification effect of *APOE4* also remained significant (β, 0.28; bootstrapped 95% CI, 0.02 to 0.53; *P* = .02; bootstrapped *P* = .048) (Figure 3C). Second, we excluded participants with AD pathology at baseline. Even with the most conservative criteria, the observed associations remained when excluding 15% abnormal Aβ42/40 (β, 0.18; bootstrapped 95% CI, 0.08 to 0.27; *P* = .001; bootstrapped *P* = .003) or p-tau_181_ values (β, 0.15; 95% CI, 0.02 to 0.28; *P* = .009; bootstrapped *P* = .02) (Figure 3D). We found a trend-level association after excluding 15% abnormal p-tau_217_ values which equaled a conservative cut point of 0.37 pg/mL (β, 0.09; bootstrapped 95% CI, −0.01 to 0.19; *P* = .04; bootstrapped *P* = .06). The association remained after applying the previously validated p-tau_217_ cut point of 0.63 pg/mL^42^ (β, 0.13; bootstrapped 95% CI, 0.02 to 0.23; *P* = .02; bootstrapped *P* = .02). Again, the modification effect of *APOE4* remained after excluding abnormal Aβ42/40 (β, 0.49; bootstrapped 95% CI, 0.17 to 0.75; *P* < .001; bootstrapped *P* = .004) or p-tau_181_ (β, 0.40; bootstrapped 95% CI, 0.02 to 0.72; *P* = .002; bootstrapped *P* = .04). The modification lost strength upon excluding abnormal p-tau_217_ values (15% highest values: β, 0.19; bootstrapped 95% CI, −0.19 to 0.56; *P* = .12; bootstrapped *P* = .33; 0.63 pg/mL: β, 0.38; bootstrapped 95% CI, 0.06 to 0.66; *P* = .003; bootstrapped *P* = .03). All results can be found in eTable 5 and eFigure 2 in Supplement 1.

## Discussion

This cohort study investigated the association between objective and self-reported measures of sleep, 24-hour activity rhythms, and Aβ PET deposition 7.8 years later in 319 participants without dementia from the prospective population-based Rotterdam Study. A more fragmented 24-hour activity rhythm based on actigraphy was associated with a higher Aβ burden at follow-up. *APOE* genotype was a significant effect modifier, such that this association was mainly seen in *APOE4* carriers. The associations remained after excluding participants with AD pathology at baseline, indicating these rhythm disturbances are likely to precede Aβ deposition. Our findings support the hypothesis that disrupted 24-hour activity rhythms could be a potential risk factor for Aβ pathology, especially in *APOE*4 carriers.

To our knowledge, only 2 previous studies investigated whether disrupted 24-hour activity rhythms are associated with Aβ pathology. Our results are consistent with a cross-sectional actigraphy-PET study by Musiek et al,^24^ which found a more fragmented 24-hour activity rhythm in 186 amyloid-positive vs negative participants of the Knight Alzheimer Disease Research Center. In contrast, no association was found between 24-hour activity rhythms and Aβ pathology in an actigraphy-PET subgroup analysis of the A4 Study,^22^ which had, however, limited statistical power (n = 59). Measuring state-of-the-art AD plasma markers at baseline allowed us to go beyond previous studies and to investigate the temporal association. Aβ42/40 and p-tau_217_ are currently the best-performing blood markers for predicting longitudinal Aβ accumulation on PET^43^ and plaques on histopathology, yielding high accuracy (area under the receiver operating characteristic curve, 0.89; 95% CI, 0.82 to 0.96).^44^ Previous work has shown that p-tau_217_ cut points are highly concordant with Aβ PET positivity and reproducible across cohorts.^42,45^ After excluding participants with abnormal Aβ42/40 or p-tau_217_, the association between a fragmented 24-hour activity rhythm and Aβ PET largely remained. Although a longitudinal actigraphy-PET study is necessary to draw causal conclusions, our results suggest that a fragmented 24-hour activity rhythm may precede Aβ accumulation and, thus, may be a risk factor of Aβ pathology.

Along the same lines, 2 prospective actigraphy studies concluded that disrupted rest-activity rhythms seem to precede symptom onset in AD.^3,5^ Monitoring 737 participants from the Rush Memory and Aging Project over 6 years, Lim et al^3^ found that individuals with a higher sleep fragmentation at baseline had a 1.5-fold risk of developing AD. Similarly, lower rest-activity rhythm amplitude and robustness increased the likelihood of developing mild cognitive impairment and dementia after 5 years of follow-up in 1282 women from the Study of Osteoporotic Fractures cohort.^5^ In contrast, an earlier investigation from the Rotterdam Study by Lysen et al,^46^ which monitored 1322 participants for up to 11 years, found no associations between fragmented 24-hour activity rhythms and dementia risk. Instead, disturbed sleep was associated with an increased risk of dementia, especially in *APOE4* noncarriers. These different conclusions should be seen as complementary rather than contradictory. Disturbed 24-hour activity rhythms and sleep may be differentially associated with the many underlying pathologies of dementia. The fact that Lysen et al^46^ found larger effect sizes in *APOE4* noncarrier suggests that they identified risk factors for dementia-related pathologies other than Aβ, which is strongly influenced by the *APOE4* allele.

We found no robust association between sleep duration and Aβ pathology. There are many previous studies on this, but the results are inconsistent. Self-reports of shorter sleep duration were associated with increased Aβ pathology in most^11,12,13,14,20^ but not all studies.^15^ However, this association has not been confirmed in previous actigraphy studies,^11,12,13,14,47^ consistent with the current findings. Different sample sizes are likely one underlying reason; actigraphy studies are typically smaller because they are more burdensome (average n = 107^15,17,18^) than self-reports. The largest study using self-reports (n = 4417) found a 0.01 increase in Aβ PET SUVR per 1 hour of less sleep,^12^ whereas we found a 0.02 increase per 1 hour of less objectively measured sleep. Therefore, it seems possible that less night-time sleep is associated with more Aβ pathology, although the effect is likely to be small and can only be robustly estimated in large samples. Inconsistencies in the literature may also be due to the fact that objective and self-reported sleep duration capture different aspects of sleep health.^11,12,15,48,49^ Self-reports reflect the experienced sleep duration, which is likely influenced by other sleep parameters (eg, how rested a person felt) and more general factors like a person’s perceived health status.^48^ It is therefore important to investigate associations with both objective and self-reported sleep measures and Aβ pathology in the same participants, as we did here.

No consensus has been reached regarding the role of *APOE4* in the relationship between sleep, 24-hour activity rhythms, and Aβ pathology. Our data suggest that *APOE4* carriers may be more susceptible to disturbances in the rest-activity rhythms. Yet, the previous 2 related studies did not consider *APOE4* as a covariate or effect modifier.^22,24^ Some indirect support for the notion of *APOE4* susceptibility comes from the Chinese Alzheimer Biomarker and Lifestyle (CABLE) study^14^ (n = 736), in which lower sleep efficiency was associated with more abnormal cerebrospinal fluid Aβ42/40 levels in *APOE4* carriers than in noncarriers. Yet, other studies did not observe such an effect.^15,24,50^ As *APOE4* noncarriers accumulate Aβ pathology much later in life than carriers,^51^ we cannot exclude that rest-activity disturbances are also detrimental in noncarriers at older ages than those studied here. While the evidence in humans remains inconclusive at this point, initial studies in AD mouse models suggest that *APOE* genotype acts as an effect modifier, leading to higher Aβ deposition following sleep deprivation, but only in the presence of human E4 and not E3.^30^

Two mechanisms by which 24-hour activity fragmentation may contribute to Aβ accumulation have been described in the literature.^7^ First, soluble Aβ is released during synaptic activity which is higher during wake than sleep.^8,52,53^ A fragmented 24-hour activity rhythm may lead to reduced periods of uninterrupted sleep which could increase neuronal activity and, therefore, a relative excess of soluble Aβ. Over time, higher soluble Aβ levels increase the likelihood that Aβ oligomers aggregate into insoluble Aβ plaques. Second, a fragmented 24-hour activity rhythm may affect Aβ clearance through the glymphatic system. The brain drains toxins such as Aβ through a dynamic interaction between interstitial fluid and cerebrospinal fluid.^54^ While the full complexity is not understood, many studies have shown that this process is more effective during sleep.^9^ One hypothesis is that the interstitial space between brain cells^9^ and the cerebrospinal fluid flow^55^ increase during sleep. Importantly, glymphatic system impairments have been reported to precede substantial Aβ accumulation in mice.^56^ Together, there is solid evidence linking the rest-activity rhythm to Aβ production and clearance, while any alteration to the rhythms, such as those shown here, could potentially contribute to AD.

### Limitations

This study has limitations. First, participants only had 1 PET scan. Hence, we could not perform longitudinal analyses limiting our ability to evaluate a potential causal association between sleep, 24-hour activity rhythms, and Aβ. We tried to circumvent this limitation by using AD plasma markers at baseline. Second, the gold standard for measuring sleep is polysomnography, which has its own limitations, such as time and cost, making polysomnography impractical for large studies. Actigraphy is less burdensome and shows fair associations with polysomnography.^57^ Third, possible sleep apnea was assessed through self-reports, and therefore we cannot entirely exclude that the associations we found were at least partially due to sleep apnea unknown to the participant.^58,59^

## Conclusions

In this study, fragmentation of the 24-hour activity rhythm was associated with a higher Aβ burden 7.8 years later, especially in *APOE4* carriers. As further evidence for this association accumulates, intervention trials will be needed to investigate whether reducing fragmentation of the rest-activity rhythms can prevent or slow AD progression.

## Notes

Supplement 1. eMethods 1. Calculation of bootstrapped confidence intervals and p-values eMethods 2. Assessment of covariates eTable 1. Sample characteristics of APOE4 carriers versus non-carriers eTable 2. Associations of 24-hour activity rhythm, sleep and Aβ positivity eTable 3. Non-linear relationship between sleep measures and Aβ pathology eTable 4. Interaction between 24h activity rhythm/sleep and APOE4 on Aβ pathology eTable 5. Stability of main results after excluding participants with AD pathology at baseline eFigure 1. Correlations between objective and self-reported sleep measures eFigure 2. Stability of main results after excluding participants with AD pathology at baseline

Supplement 2. Data sharing statement
