# Supplementary material for: Sleep, 24-Hour Activity Rhythms, and Subsequent Amyloid-β Pathology
Source: JAMA Neurol. 2024 Jun 24;81(8):824–34. doi: 10.1001/jamaneurol.2024.1755 (PMC11197458; doi:10.1001/jamaneurol.2024.1755)
Supplement: Supplement 1. — eMethods 1. Calculation of bootstrapped confidence intervals and p-values eMethods 2. Assessment of covariates eTable 1. Sample characteristics of APOE4 carriers versus non-carriers eTable 2. Associations of 24-hour activity rhythm, sleep and Aβ positivity eTable 3. Non-linear relationship between sleep measures and Aβ pathology eTable 4. Interaction between 24h activity rhythm/sleep and APOE4 on Aβ pathology eTable 5. Stability of main results after excluding participants with AD pathology at baseline eFigure 1. Correlations between objective and self-reported sleep measures eFigure 2. Stability of main results after excluding participants with AD pathology at baseline [file jamaneurol-e241755-s001.pdf]

Supplemental Online Content

Nguyen Ho PT, Hoepel SJW, Rodriguez-Ayllon M, Luik AI, Vernooij MW, Neitzel J. Sleep, 24-hour activity rhythms, and subsequent amyloid-β pathology. *JAMA Neurol*. Published online June 24, 2024. doi:10.1001/jamaneurol.2024.1755

eMethods 1. Calculation of bootstrapped confidence intervals and p-values
eMethods 2. Assessment of covariates
eTable 1. Sample characteristics of APOE4 carriers versus non-carriers
eTable 2. Associations of 24-hour activity rhythm, sleep and A□ positivity
eTable 3. Non-linear relationship between sleep measures and A□ pathology
eTable 4. Interaction between 24h activity rhythm/sleep and APOE4 on A□ pathology
eTable 5. Stability of main results after excluding participants with AD pathology at baseline
eFigure 1. Correlations between objective and self-reported sleep measures
eFigure 2. Stability of main results after excluding participants with AD pathology at baseline

This supplemental material has been provided by the authors to give readers additional information about their work.

© 2024 Nguyen Ho PT et al. *JAMA Neurology.*

eMethods 1. Calculation of bootstrapped confidence intervals and p-values

We used non-parametric bootstrapping with 1,000 replications to calculate 95% confidence intervals and p-values for our linear regression models. To this end, we randomly generated 1,000 new datasets from the original dataset by resampling with replacement using the boot function (v1.3-28) in R. That means that some values will occur more often than in the original dataset, others less often, and some may not occur at all in the new datasets. We next fitted our linear regression models to all new datasets and computed regression coefficients (beta-values) and the corresponding test statistic (t-values). 95% confidence intervals were computed as the 2,5th and 97,5th quantiles of the 1,000 bootstrapped beta-values (see example in figure on the right).

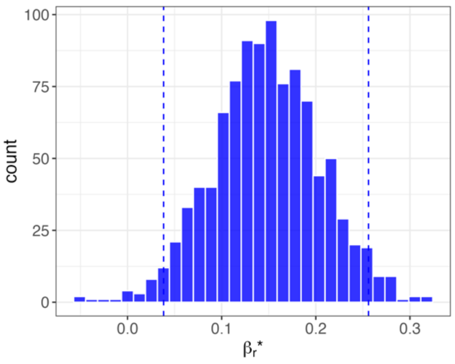

For hypothesis testing, we generated a null-distribution (grey histogram in left figure) of the test statistic by subtracting each bootstrapped t-value (blue histogram) by the mean of all t-values of the bootstrap.

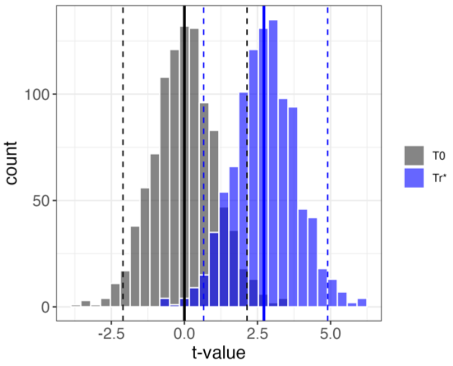

$$T_0^* = T_r^* - \overline{T_r^*} \quad (1)$$

We obtained two-sided p-values by calculating the proportion of cases where the t-value in the new datasets exceeded the t-value in our original sample. This proportion is a p-value as it expresses the probability of observing a t-value of at least this magnitude if the null hypothesis is true. We derived the formula from Davison and Hinkley (1997), *Bootstrap Methods and their Application*, p. 141:

$$p_{boot} = \frac{\{|T_0^*| \geq |T_1|\} + 1}{R+1} \quad (2)$$

Where T_1 = t-value from the original dataset, T_0^* = bootstrapped null distribution from (1), R = number of replications

© 2024 Nguyen Ho PT et al. *JAMA Neurology.*

eMethods 2. Assessment of covariates

Covariates were collected from home interviews, medical examinations, or the sleep diary at the baseline visit [1]. APOE genotyping was determined by a biallelic TaqMan assay (rs7412 and rs429358; Applied Biosystems). Hypertension was defined as a systolic blood pressure ≥140 and/or a diastolic blood pressure ≥90 and/or taking hypertensive medications. Diabetes was defined as fasting serum glucose levels ≥7.0 mmol/l, non-fasting serum glucose levels ≥11.0 mmol/l (if fasting samples were unavailable) and/or the use of blood glucose-lowering medication. Participants were categorized as having possible sleep apnea when they reported breathing pauses at least one to two nights per week or reported breathing pauses occasionally and snoring at least two nights per week on the Pittsburgh sleep quality index. As part of the sleep diary, participants were asked if they used any sleep medication each night. Physical activity was acquired using the LASA Physical Activity Questionnaire. Depressive symptoms were assessed with the Center for Epidemiologic Studies Depression scale. Any missing values were imputed using the Multiple Imputation by Chained Equation (MICE) method [2].

© 2024 Nguyen Ho PT et al. *JAMA Neurology.*

eTable 1. Sample characteristics of APOE4 carriers versus non-carriers

Variables	Levels	APOE4 non-carrier	APOE4 carrier	p
n		229	90	
**Demographic information**				
Age at sleep assessment, mean (SD)		61.81 (5.54)	60.59 (4.95)	0.069
Age at PET, mean (SD)		69.56 (5.42)	68.41 (4.73)	0.079
Years between sleep assessment and PET, mean (SD)		7.75 (2.33)	7.82 (2.53)	0.799
Sex (%)	Female	105 (45.9)	45 (50.0)	0.587
	Male	124 (54.1)	45 (50.0)	
Education (%)	Primary	14 (6.1)	7 (7.8)	0.763
	Lower	67 (29.3)	22 (24.4)	
	Intermediate	60 (26.2)	27 (30.0)	
	Higher	88 (38.4)	34 (37.8)	
Paid employment (%)	No	105 (53.0)	31 (41.3)	0.112
	Yes	93 (47.0)	44 (58.7)	
MMSE at sleep assessment, mean (SD)		28.60 (1.37)	28.41 (1.13)	0.285
**Genetic measures**				
Ancestry (%)	African	1 (0.4)	1 (1.1)	0.901
	Asian	3 (1.3)	1 (1.1)	
	European	202 (88.2)	78 (86.7)	
	Mixed	23 (10.0)	10 (11.1)	
**Amyloid PET measures**				
Amyloid PET status (%)	Negative	211 (92.1)	59 (65.6)	<0.001
	Positive	18 (7.9)	31 (34.4)	
SUVR, mean (SD)		1.00 (0.12)	1.11 (0.19)	<0.001
**24-hour activity rhythm measures from actigraphy**				
Interdaily stability, mean (SD)		0.73 (0.11)	0.72 (0.12)	0.267
Intradaily variability, mean (SD)		0.46 (0.15)	0.45 (0.12)	0.499
L5 start time (hh:mm), mean (SD)		25.32 (1.14)	25.28 (1.29)	0.764
**Objective sleep measures (actigraphy)**				
Total sleep time (hours), mean (SD)		6.12 (0.90)	6.22 (0.76)	0.374
Sleep latency (minutes), mean (SD)		18.12 (17.23)	15.90 (13.77)	0.276
Sleep efficiency (%), mean (SD)		75.56 (8.14)	78.37 (6.92)	0.004
Wake after sleep onset (minutes), mean (SD)		56.07 (23.48)	48.81 (18.75)	0.009
**Subjective sleep measures (sleep diaries)**				
Total sleep time - diary (hours), mean (SD)		6.70 (0.93)	6.82 (0.81)	0.275
Time in bed - diary (hours), mean (SD)		8.12 (0.85)	7.96 (0.81)	0.114

© 2024 Nguyen Ho PT et al. *JAMA Neurology.*

Sleep latency - diary (minutes), mean (SD)		23.00 (21.57)	20.04 (14.99)	0.235
Sleep efficiency - diary (%), mean (SD)		83.10 (10.51)	85.58 (7.99)	0.044
Sleep quality - diary, mean (SD)		5.34 (1.61)	5.79 (1.38)	0.022
Napping - diary, mean (SD)		1.01 (1.68)	0.72 (1.21)	0.133
Daytime sleepiness (days/week), mean (SD)		0.75 (1.30)	0.46 (0.95)	0.056
**Others**				
Type of actigraph (%)	Actiwatch	175 (76.4)	78 (86.7)	0.060
	GENEActiv	54 (23.6)	12 (13.3)	
Sleep apnea (%)	No	176 (87.6)	67 (84.8)	0.677
	Yes	25 (12.4)	12 (15.2)	
Sleep medication (%)	No	202 (88.2)	80 (88.9)	1.000
	Yes	27 (11.8)	10 (11.1)	
BMI, mean (SD)		27.39 (4.03)	27.07 (3.41)	0.547
Hypertension (%)	No	93 (40.6)	48 (53.3)	0.053
	Yes	136 (59.4)	42 (46.7)	
Diabetes (%)	No	209 (91.3)	83 (93.3)	0.723
	Yes	20 (8.7)	6 (6.7)	
Smoking (%)	No	166 (81.8)	58 (77.3)	0.509
	Yes	37 (18.2)	17 (22.7)	
Physical activity (MET hours/week), mean (SD)		62.53 (56.47)	52.85 (43.13)	0.158
Depressive symptoms, mean (SD)		4.79 (6.55)	2.97 (5.12)	0.019

© 2024 Nguyen Ho PT et al. *JAMA Neurology.*

eTable 2. Associations of 24-hour activity rhythm, sleep and Aβ positivity

Variable	Missing data	Model 1			Model 2		
		OR (95% CI)	p-value	p-value [fdr]	OR (95% CI)	p-value	p-value [fdr]
**24-hour activity rhythm measures (actigraphy)**							
Interdaily stability	9	1.07 [0.74,1.60]	0.727	0.998	1.20 [0.79,1.87]	0.408	0.953
Intradaily variability	9	1.36 [0.94,1.93]	0.095	0.664	1.38 [0.93,2.05]	0.106	0.741
L5 start time	11	1.06 [0.73,1.51]	0.762	0.998	0.99 [0.67,1.47]	0.979	0.979
**Objective sleep measures (actigraphy)**							
Total sleep time	0	0.98 [0.69,1.40]	0.904	0.998	1.02 [0.70,1.50]	0.916	0.979
Sleep latency	0	0.95 [0.63,1.40]	0.810	0.998	0.93 [0.61,1.38]	0.716	0.979
Sleep efficiency	0	1.00 [0.71,1.43]	0.998	0.998	1.03 [0.71,1.50]	0.895	0.979
Wake after sleep onset	0	1.20 [0.81,1.74]	0.342	0.998	1.19 [0.78,1.78]	0.397	0.953
**Subjective sleep measures (sleep diary)**							
Total sleep time	0	1.22 [0.86,1.76]	0.263	0.460	1.29 [0.89,1.91]	0.189	0.441
Time in bed	0	1.01 [0.70,1.45]	0.978	0.990	1.03 [0.70,1.53]	0.875	0.875
Sleep latency	2	1.00 [0.68,1.42]	0.990	0.990	1.04 [0.68,1.51]	0.846	0.875
Sleep efficiency	0	1.39 [0.94,2.13]	0.113	0.409	1.40 [0.93,2.18]	0.118	0.412
Sleep quality	0	1.44 [0.94,2.35]	0.117	0.409	1.50 [0.94,2.53]	0.105	0.412
Napping	0	0.75 [0.47,1.13]	0.204	0.460	0.76 [0.46,1.19]	0.264	0.461
Daytime sleepiness	0	0.92 [0.57,1.39]	0.719	0.990	0.95 [0.56,1.50]	0.843	0.875

© 2024 Nguyen Ho PT et al. *JAMA Neurology.*

eTable 3. Non-linear relationship between sleep measures and Aβ pathology burden (SUVR)

Variable	Missing data	Model 1				Model 2			
		β [95% CI]	p	p [boot]	p [fdr]	β [95% CI]	p	p [boot]	p [fdr]
Total sleep time, short - optimal	3	-0.11 [-0.32, 0.11]	0.281	0.293	0.375	-0.08 [-0.28, 0.14]	0.451	0.446	0.602
Total sleep time squared, hours	0	-0.10 [-0.19,-0.01]	0.056	0.034	0.168	-0.08 [-0.17, 0.01]	0.133	0.081	0.365
Total in bed squared, hours	0	-0.09 [-0.19, 0.02]	0.084	0.094	0.168	-0.07 [-0.17, 0.03]	0.182	0.165	0.365
L5 start time squared, hh:mm	11	0.00 [-0.10, 0.09]	0.929	0.930	0.929	-0.02 [-0.11, 0.08]	0.749	0.724	0.749

© 2024 Nguyen Ho PT et al. *JAMA Neurology.*

eTable 4. Interaction between 24h activity rhythm/sleep and *APOE4* on Aβ pathology burden (SUVR)

Variable	Stratified analyses										Interaction analyses								
	APOE4 carrier					APOE4 non-carrier					Model 1					Model 2			
	n	ß [95% CI]	p	p [boot]	p [fdr]	n	ß [95% CI]	p	p [boot]	p [fdr]	Missing data	ß [95% CI]	p	p [boot]	p [fdr]	ß [95% CI]	p	p [boot]	p [fdr]
**24-hour activity rhythm measures (actigraphy)**																			
Interdaily stability	88	0.01 [-0.19, 0.24]	0.916	0.888	0.916	222	0.05 [-0.03, 0.14]	0.365	0.247	0.728	9	-0.04 [-0.25,0.20]	0.739	0.723	0.808	-0.01 [-0.24,0.23]	0.935	0.933	0.960
Intradaily variability	88	0.38 [0.05, 0.64]	0.016	0.028	0.113	222	0.07 [-0.04, 0.18]	0.161	0.189	0.451	9	0.38 [0.10,0.66]	0.002	0.021	0.013	0.37 [0.07,0.63]	0.003	0.026	0.020
L5 start time	87	0.02 [-0.22, 0.25]	0.879	0.868	0.916	221	-0.04 [-0.13, 0.07]	0.468	0.444	0.728	11	0.10 [-0.16,0.34]	0.373	0.389	0.653	0.09 [-0.16,0.35]	0.453	0.468	0.792
**Objective sleep measures (actigraphy)**																			
Total sleep time	90	0.03 [-0.23, 0.33]	0.831	0.799	0.916	229	-0.13 [-0.22,-0.05]	0.010	0.009	0.113	0	0.11 [-0.16,0.42]	0.364	0.457	0.653	0.12 [-0.15,0.44]	0.332	0.417	0.774
Sleep latency	90	0.20 [0.00, 0.45]	0.135	0.065	0.451	229	-0.09 [-0.17,-0.01]	0.072	0.017	0.335	0	0.26 [0.02,0.49]	0.030	0.020	0.106	0.26 [0.00,0.49]	0.032	0.032	0.112
Sleep efficiency	90	-0.03 [-0.29, 0.22]	0.825	0.807	0.916	229	-0.05 [-0.15, 0.05]	0.331	0.370	0.728	0	0.03 [-0.24,0.32]	0.798	0.802	0.808	0.01 [-0.25,0.30]	0.960	0.965	0.960
Wake after sleep onset	90	0.12 [-0.25, 0.43]	0.434	0.519	0.728	229	0.01 [-0.10, 0.11]	0.840	0.857	0.916	0	0.03 [-0.32,0.37]	0.808	0.861	0.808	0.03 [-0.33,0.36]	0.795	0.858	0.960
**Subjective sleep measures (sleep diary)**																			
Total sleep time	90	0.15 [-0.12, 0.46]	0.301	0.296	0.662	229	-0.08 [-0.17, 0.00]	0.125	0.071	0.662	0	0.16 [-0.10,0.47]	0.193	0.282	0.367	0.18 [-0.08,0.50]	0.153	0.224	0.269
Time in bed	90	0.08 [-0.21, 0.38]	0.543	0.596	0.662	229	-0.14 [-0.23,-0.04]	0.009	0.006	0.126	0	0.15 [-0.14,0.48]	0.210	0.343	0.367	0.17 [-0.12,0.50]	0.144	0.275	0.269
Sleep latency	90	-0.14 [-0.51, 0.16]	0.371	0.403	0.662	227	0.03 [-0.07, 0.15]	0.556	0.601	0.662	2	-0.20 [-0.58,0.16]	0.131	0.290	0.367	-0.19 [-0.60,0.16]	0.152	0.326	0.269
Sleep efficiency	90	0.12 [-0.18, 0.45]	0.444	0.452	0.662	229	0.03 [-0.07, 0.12]	0.568	0.548	0.662	0	0.08 [-0.26,0.45]	0.520	0.634	0.528	0.06 [-0.30,0.41]	0.646	0.709	0.646
Sleep quality	90	0.17 [-0.08, 0.44]	0.292	0.195	0.662	229	0.00 [-0.10, 0.09]	0.982	0.983	0.982	0	0.26 [0.00,0.53]	0.038	0.050	0.263	0.20 [-0.07,0.50]	0.114	0.146	0.269
Napping	90	-0.17 [-0.50, 0.12]	0.327	0.250	0.662	229	-0.05 [-0.14, 0.04]	0.324	0.240	0.662	0	-0.09 [-0.42,0.20]	0.528	0.553	0.528	-0.10 [-0.43,0.18]	0.487	0.497	0.646
Daytime sleepiness	90	-0.05 [-0.35, 0.24]	0.739	0.671	0.796	229	0.03 [-0.04, 0.11]	0.508	0.420	0.662	0	-0.13 [-0.40,0.16]	0.342	0.326	0.479	-0.08 [-0.34,0.25]	0.567	0.553	0.646

© 2024 Nguyen Ho PT et al. *JAMA Neurology.*

eTable 5. Stability of main results after excluding participants with AD pathology at baseline

Determinant	Cutoff variable	Cutoff	n	Model 1			Model 2		
				β [95% CI]	p	p [boot]	β [95% CI]	p	p [boot]
IV	Ab4240	5%	293	0.14 [0.03,0.24]	0.013	0.015	0.13 [0.02,0.24]	0.025	0.030
IV	Ab4240	10%	277	0.15 [0.05,0.25]	0.005	0.010	0.15 [0.03,0.26]	0.007	0.011
IV	Ab4240	15%	261	0.18 [0.08,0.27]	0.001	0.003	0.17 [0.07,0.27]	0.002	0.005
IV	pTau181	5%	293	0.15 [0.04,0.26]	0.006	0.009	0.14 [0.03,0.26]	0.013	0.023
IV	pTau181	10%	278	0.17 [0.04,0.28]	0.003	0.009	0.15 [0.03,0.27]	0.011	0.020
IV	pTau181	15%	262	0.15 [0.02,0.28]	0.009	0.024	0.12 [0.00,0.25]	0.044	0.069
IV	pTau217	5%	293	0.10 [0.00,0.20]	0.057	0.063	0.09 [-0.02,0.19]	0.123	0.115
IV	pTau217	10%	278	0.10 [0.00,0.21]	0.054	0.077	0.08 [-0.02,0.18]	0.138	0.132
IV	pTau217	15%	262	0.09 [-0.01,0.19]	0.042	0.060	0.06 [-0.02,0.14]	0.174	0.168
IV	pTau217	>0.63 pg/mL	301	0.13 [0.02,0.23]	0.020	0.020	0.11 [0.00,0.21]	0.046	0.047
IV * APOE4	Ab4240	5%	293	0.33 [0.01,0.59]	0.009	0.042	0.31 [-0.02,0.57]	0.017	0.058
IV * APOE4	Ab4240	10%	277	0.47 [0.16,0.72]	0.000	0.006	0.45 [0.15,0.69]	0.000	0.009
IV * APOE4	Ab4240	15%	261	0.49 [0.17,0.75]	0.000	0.004	0.48 [0.16,0.74]	0.000	0.008
IV * APOE4	pTau181	5%	293	0.41 [0.09,0.69]	0.001	0.012	0.40 [0.08,0.67]	0.001	0.017
IV * APOE4	pTau181	10%	278	0.43 [0.08,0.70]	0.001	0.020	0.43 [0.09,0.70]	0.001	0.021
IV * APOE4	pTau181	15%	262	0.40 [0.02,0.72]	0.002	0.038	0.39 [0.03,0.70]	0.002	0.039
IV * APOE4	pTau217	5%	293	0.26 [-0.12,0.56]	0.048	0.153	0.24 [-0.13,0.53]	0.075	0.193
IV * APOE4	pTau217	10%	278	0.23 [-0.17,0.57]	0.073	0.234	0.21 [-0.20,0.53]	0.109	0.281
IV * APOE4	pTau217	15%	262	0.19 [-0.19,0.56]	0.124	0.333	0.13 [-0.23,0.49]	0.288	0.461
IV * APOE4	pTau217	>0.63 pg/mL	301	0.38 [0.06,0.66]	0.003	0.032	0.37 [0.03,0.66]	0.004	0.035

We determined whether the main effect of intradaily variability (IV), fragmentation of the 24-hour activity rhythm, and the IV-by-APOE4 interaction on Aβ PET burden remained after excluding participants with abnormal AD plasma markers (lowest Aβ42/40, highest pTau181, pTau217) at baseline (see also eFigure 2). We hypothesized that if a fragmented 24-hour activity rhythm preceded Aβ pathology, then the association should remain significant after the exclusions. We decided to exclude 5%, 10% and 15% of the most abnormal values, because Aβ PET positivity was 15.4% at follow-up, and we would therefore expect a maximum of 15% of the participants to show AD pathology at baseline. Alternatively, we used established cut-points for participant exclusion base: pTau217 positive >0.63 pg/mL, and pTau217 negative <0.40 pg/mL.

© 2024 Nguyen Ho PT et al. *JAMA Neurology.*

eFigure 1. Correlations between objective and self-reported sleep/24-hour activity rhythms measures

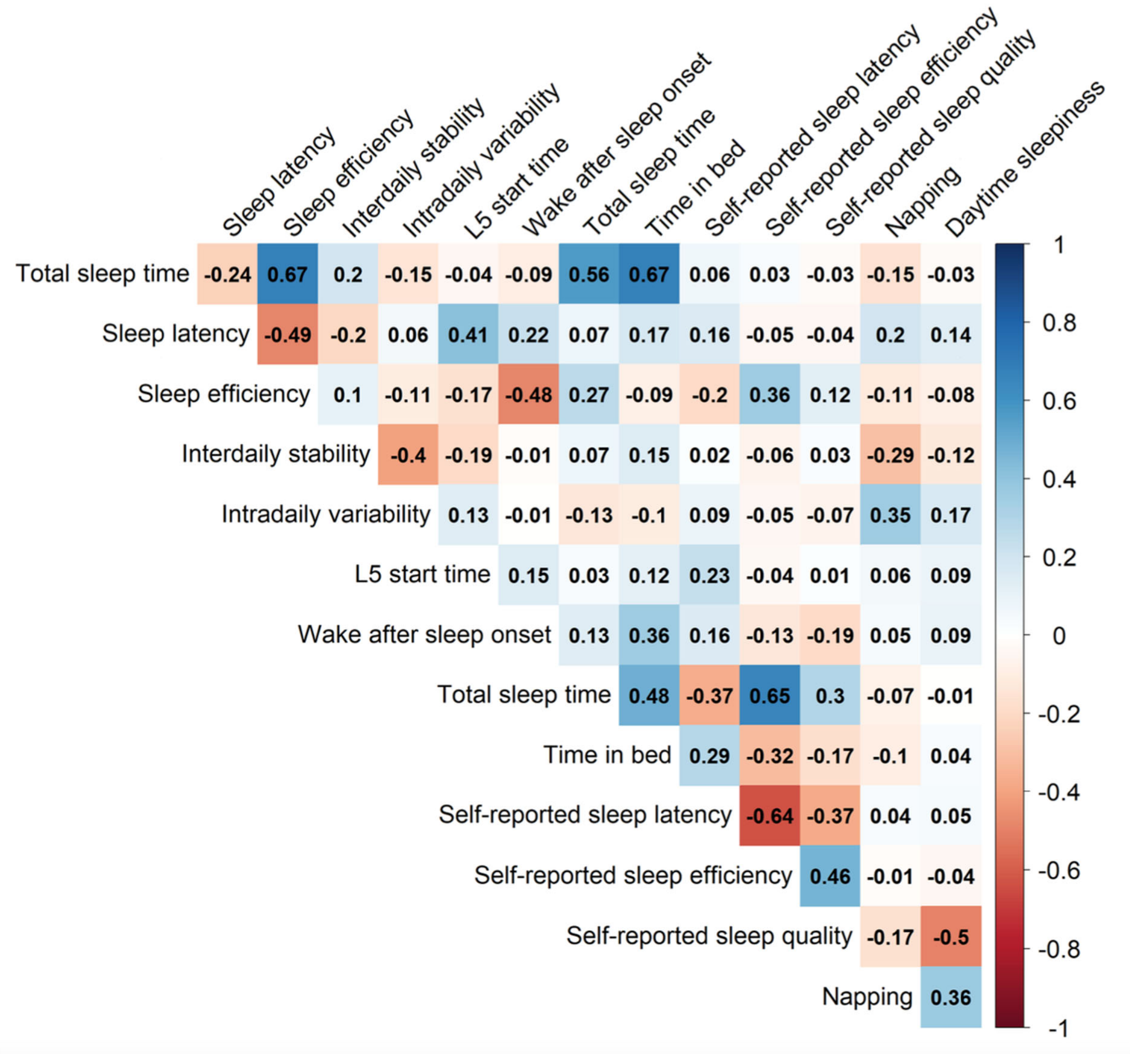

All measures which are not marked with 'Self-reported' came from actigraphy

© 2024 Nguyen Ho PT et al. *JAMA Neurology.*

eFigure 2. Stability of the main results after excluding participants with AD pathology at baseline

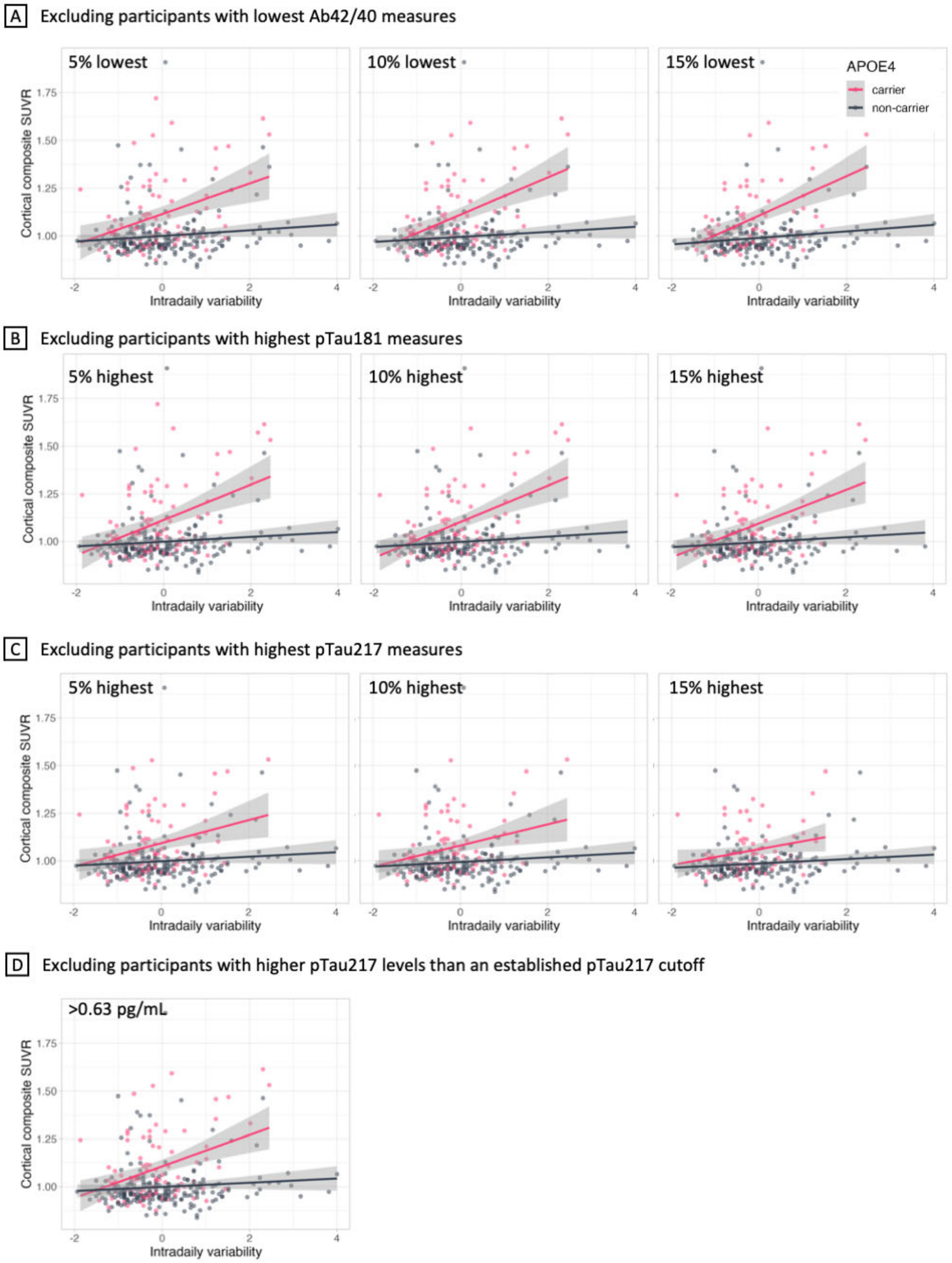

See eTable 5 for the detailed statistical results.

© 2024 Nguyen Ho PT et al. *JAMA Neurology*.

References

1. Hofman A, Rodriguez-Ayllon M, Vernooij MW, et al. Physical activity levels and brain structure in middle-aged and older adults: a bidirectional longitudinal population-based study. *Neurobiol Aging*. 2023;121:28-37. doi:10.1016/j.neurobiolaging.2022.10.002
2. Buuren S van, Groothuis-Oudshoorn K. mice : Multivariate Imputation by Chained Equations in R. *J Stat Softw*. 2011;45(3). doi:10.18637/jss.v045.i03

© 2024 Nguyen Ho PT et al. *JAMA Neurology.*
