# Supplementary material for: Sleep, 24-Hour Activity Rhythms, and Subsequent Amyloid-β Pathology
Source: JAMA Neurol. 2024 Jun 24;81(8):824–34. doi: 10.1001/jamaneurol.2024.1755 (PMC11197458; doi:10.1001/jamaneurol.2024.1755)
Supplement: Supplement 2. — Data sharing statement [file jamaneurol-e241755-s002.pdf]

Data Sharing Statement

Nguyen Ho. Sleep and 24-Hour Activity Rhythms and subsequent Amyloid-β Pathology. *JAMA Neurol*. Published June 24, 2024. doi:10.1001/jamaneurol.2024.1755

Data
Data available: No

Additional Information
Explanation for why data not available: Data can be obtained upon request. Requests should be directed to the management team of the Rotterdam Study (secretariat.epi@erasmusmc.nl), which has a protocol for approving data requests. Because of restrictions based on privacy regulations and informed consent of the participants, data cannot be made freely available in a public repository.
